# How to Survive 33 min after the Umbilical of a Saturation Diver Severed at a Depth of 90 msw?

**DOI:** 10.3390/healthcare10030453

**Published:** 2022-02-28

**Authors:** Sven Dreyer, Andreas Deussen, Dietmar Berndt, Jochen D. Schipke

**Affiliations:** 1 Hyperbaric Oxygen Therapy, University Hospital Düsseldorf, Moorenstrasse 5, 40225 Düsseldorf, Germany; sven.dreyer@med.uni-duesseldorf.de; 2 Institute of Physiology, Medical Faculty Carl Gustav Carus, TU Dresden, 01307 Dresden, Germany; andreas.deussen@tu-dresden.de; 3 Diving Equipment–Accident Investigation/Technology Evaluation, 76297 Stutensee, Germany; dietmar_berndt@t-online.de; 4 Forschungsgruppe Experimentelle Chirurgie, Universitäts-Klinikum Düsseldorf, Moorenstrasse 5, 40225 Düsseldorf, Germany

**Keywords:** saturation diving, heliox, accident, umbilical, respiratory heat loss

## Abstract

In 2012, a severe accident happened during the mission of a professional saturation diver working at a depth of 90 m in the North Sea. The dynamic positioning system of the diver support vessel crashed, and the ship drifted away from the working place, while one diver’s umbilical became snagged on a steel platform and was severed. After 33 min, he was rescued into the diving bell, without exhibiting any obvious neurological injury. In 2019, the media and a later ‘documentary’ film suggested that a miracle had happened to permit survival of the diver once his breathing gas supply was limited to only 5 min. Based on the existing data and phone calls with the diver concerned (Dc), the present case report tries to reconstruct, on rational grounds, how Dc could have survived after he was cut off from breathing gas, hot water, light and communication while 90 m deep at the bottom of the sea. Dc carried bail-out heliox (86/14) within two bottles (2 × 12 L × 300 bar: 7200 L). Calculating Dc’s varying per-minute breathing gas consumption over time, both the decreased viscosity of the helium mix and the pressure-related increase in viscosity did not exhibit a breathing gas gap. Based on the considerable respiratory heat loss, the core temperature was calculated to be as low as 28.8 °C to 27.2 °C after recovery in the diving bell. In accordance with the literature, such values would be associated with impaired or lost consciousness, respectively. Relocating Dc on the drilling template by using a remotely operated vehicle (ROV), the transport of the victim to the bell and subsequent care in the hyperbaric chamber must be regarded as exemplary. We conclude that, based on rational arguments and available literature data, Dc’s healthy survival is not a miracle, as it can be convincingly explained by means of reliable data. Remaining with a breathing gas supply sufficient for five minutes only would not have ended in a miracle but would have ended in death by suffocation. Nevertheless, survival of such an accident may appear surprising, and probably the limit for a healthy outcome was very close. We conclude, in addition, that highly effective occupational safety measures, in particular the considerable bail-out heliox reserve, secured the healthy survival. Nevertheless, the victim’s survival is likely to be due to his excellent diving training, together with many years of diving routine. The rescue action of the second diver and Dc’s retrieval by the ROV operator are also suggestive of the behavior of carefully selected crew members with the high degree of professional qualification needed to correctly function in a hostile environment.

## 1. Background

**Figure 1:**
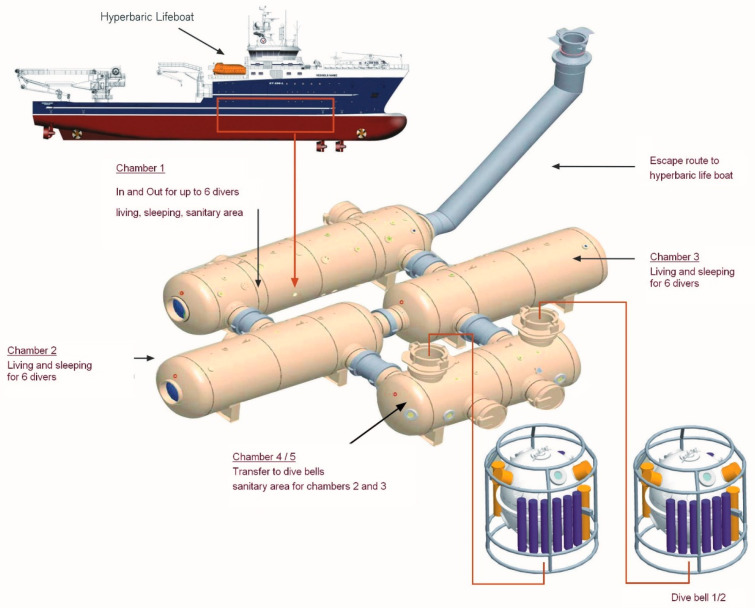
The divers’ support vessel ‘Bibby Topaz’ accommodates in its hull four hyperbaric chambers which are connected to each other. A diving bell can be connected to two of these chambers. Divers can be transported within these bells in the vicinity of the workplace. Reprinted with permission from [1]. Copyright 2006 Dräger Safety AG & Co. KgaA.

The ‘Bibby Topaz’ is a highly specialized diver support vessel with a 130-man crew. This vessel has a length of 107 m and weighs 8000 t (Figure 1). These types of vessels assist off-shore activities of the oil and gas industries. Among other things, repair work and inspections of underwater constructions are performed. As such works are performed at considerable depths and typically last for hours, saturation divers are employed, i.e., the entire body stays helium-saturated during a mission lasting for some weeks.

Based on existing data, the present case report tries to reconstruct, how this diver could have survived for 33 min with no neurological injury after his umbilical was severed and he was cut off from breathing gas, hot water, light and communication at 90 m depth at the bottom of the sea.

### 1.1. The Case

On 18 September 2012, Bibby Topaz arrived at its destination, an oil field in the North Sea, 200 km east of Aberdeen. The dynamic positioning (DP) system was activated to keep the Topaz immediately above the working site, a steel template at a depth of 90 m. Meanwhile, the wind had reached 7 bft, and waves had reached a height of 4 m. These and the following data are listed in a reconstruction of the incident [2].

Three divers that formed the first operation team of four in total entered one of the hyperbaric chambers: the diver concerned (Dc), diver2 (D2), and diver3. The latter would later stay in the diving bell (the bellman). The divers were compressed in accordance with the pressure of their working destination. Due to the large proportion of helium, more than 90%, in the breathing gas within the hyperbaric chambers, the voice—due to resonance shifts in the vocal cavities—starts to squeal, known as the Donald-Duck effect. In these chambers, the minimum pO_2_ should not be below 0.4 bar, while for the bail-out gas this should be at a maximum of 1.4 bar. Thus, for a depth of 90 m, heliox 96/14 was contained in the bail-out bottles [3].

**Figure 2:**
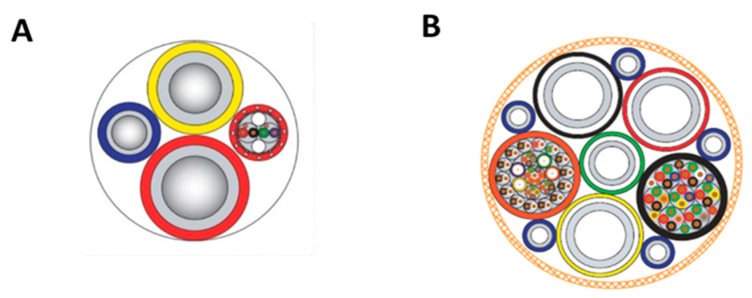
Examples of umbilical cross sections. Different supply lines exist for different purposes. (**A**): Umbilical from diving bell to diver (Ø: ~4.5 cm). (**B**): Umbilical from diver’s support vessel to diving bell (Ø: ~6 cm). Reprinted with permission from Miller [4]. Copyright 2021 Bay-Tech Industries.

**Figure 3:**
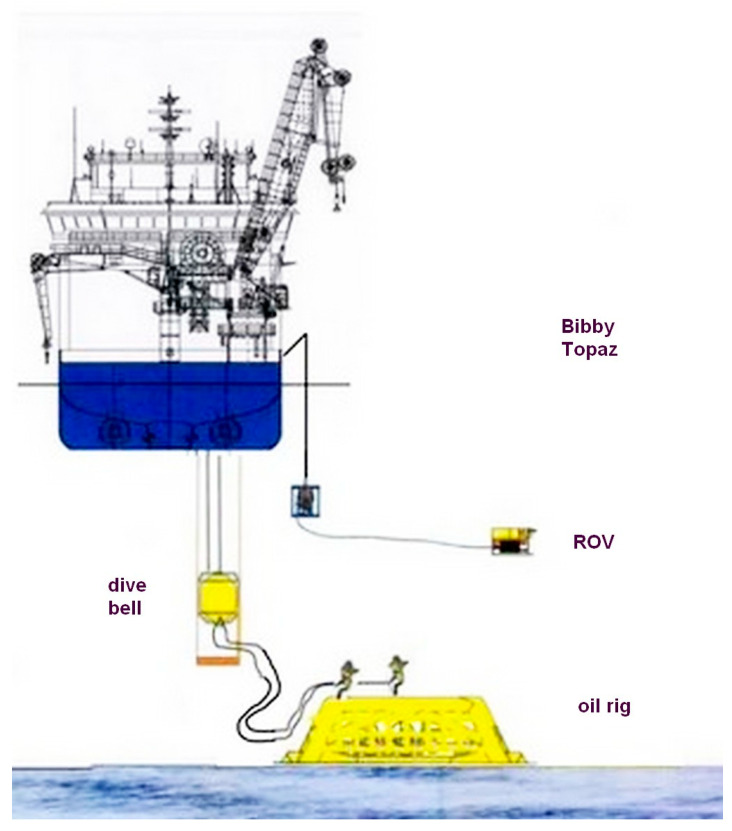
Works on an oil rig. The divers are supplied from a diving bell that in turn is supplied by a diver support vessel (see Figure 2). The divers are surveyed by a remotely-operated vehicle (ROV). Reprinted with permission from [5]. Copyright 2012 slideshare.net.

The three divers entered the diving bell that was undocked at 20.13 pm. Eighteen minutes later, the divers reached the planned depth, 10 m above their workplace. After a further 15 min, Dc and D2 left the bell saying *‘See you in six hours’*, the length of a shift. Both divers stayed connected to the bell via their umbilicals (Figure 2) of 27 m length. Three minutes later, both divers started maintenance work on pipe plugs and a steel template. During their work, they were surveyed by a remotely operated vehicle (ROV) (Figure 3).

After about 20 min, the computer responsible for the DP system, crashed and an alarm was triggered. Due to strong winds and heavy seas, the ‘Topaz’ started drifting away from its position. Thus, the bellman ordered the two divers to return to the diving bell. Along his umbilical, D2 arrived safely at the bell, while Dc tried to follow. However, his umbilical became snagged on a part of the steel construction. While the 8000 t ‘Topaz’ continued drifting away, the umbilical became an anchor line and finally severed (22.13 o’clock). From then on, Dc found himself alone. He was separated from breathing gas, hot water to heat his suit, communication with the ‘Topaz’, and light, and was therefore without orientation. He opened a valve in his helmet to access to bail-out heliox supply (two bottles; 12 L each; 300 bar).

**Figure 4:**
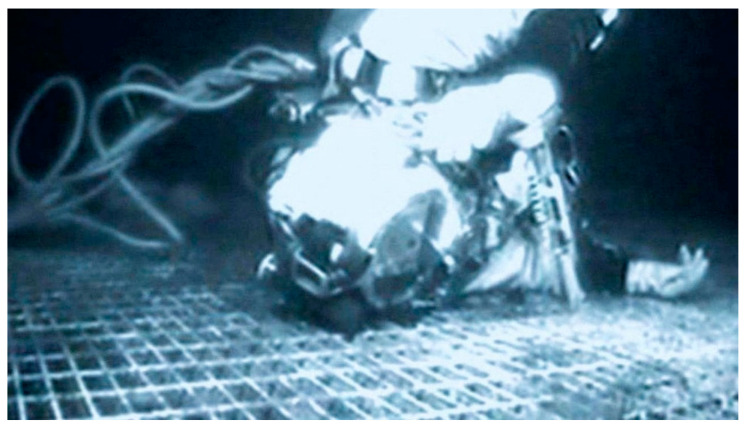
The remotely operating vehicle (ROV) finds Dc on top of the steel construction. Reprinted with permission from [6]. Copyright 2014 Floating Harbour.

Just 20 min later, the DP-system computer was successfully rebooted and had started functioning properly. In the meantime, the ‘Topaz’ had drifted 240 m away from its former position. The ROV found Dc laying on top of the steel construction (Figure 4). The images confirmed that Dc was alive, because he waved to the camera and its lights. Bubbles in the vicinity of the helmet were visible.

After the ‘Topaz’ had reached her initial position, D2 left the diving bell to dive to the steel construction, situated only 18 m from the bell. He found Dc, who was motionless in the meantime, and transported him to the bell with its warm heliox atmosphere.

At 22.46 pm, i.e., 33 min after the umbilical was severed, Dc’s helmet was removed. He was unconscious, his face blue, and with slow and shallow breathing. After D2 had delivered two deep mouth-to-mouth breaths, Dc’s respiration became more physiologically normal and vital signs became stable after only 18 min.

After the diving bell was locked, it was hauled up to the supply vessel. One hour after Dc was rescued into the bell, the bell docked to the hyperbaric chamber in the hull of ‘Topaz’. At 23.38 pm, Dc, with the assistance of D2 and the bellman, climbed out of the bell into the chamber below Finally, the ‘Topaz’ returned home at around midnight, after the ROV was back aboard the vessel.

In the meantime, Dc was further warmed in his bed with blankets. A heating mat was placed on his front. The core temperature increased, and liquid was given orally. The remote consulting physician at the Capita Health Center in Aberdeen was pleased with his progress. Dc had reached a stable condition at 2 o’clock and would be monitored each half hour, and later each full hour. At 7.45 am, decompression for all twelve Topaz divers commenced [5].

After a 15-h return trip, the ‘Topaz’ docked at the Aberdeen harbor. At 15.00 pm, the diving physician came on board and talked with Dc, who was still decompressing in the hyperbaric chamber.

The decompression was completed on the 22 September 2012, i.e., after 78 h, helium had been largely cleared from the body. Dc and the physician could now communicate with each other directly. Final examinations in the Capita Health Center exhibited no, and in particular no neurological, injury. The SAT, a test of the cognitive and analytic properties of students that apply for admission to universities, was passed by Dc with no difficulties.

Three weeks later, Dc was to be found on a new diving mission, just after his marriage. His new wife did not want to talk Dc out of his dream profession.

### 1.2. The Miracle

‘Miracle of diver who cheated death when stranded 300ft under with NO OXYGEN for 30 minutes’ was the headline of the British newspaper ‘Express’ at the time [7]. ‘Underwater Current’ contributed in a very similar manner: ‘He Was 330 Feet Deep Without Anything to Breathe—and Survived’ [8].

This case report attempts to reconstruct the circumstances of this diving accident and the emergency care, and thus to make the positive outcome rationally understandable, and to propose potential improvements for emergency systems in diving accidents.

After the umbilical severed, Dc had to rely on the heliox supply in his reserve bottles. In the documentary film about his accident, during television interviews and in print media, people repeatedly asked for an explanation of how Dc was able to survive for 33 min when the heliox supply was only supposed to last for 5 min.

The commonly accepted assumption was that irreversible brain damage occurs after only 3 to 5 min in the case of oxygen deprivation [9]. However, it is equally astonishing and pleasing that Dc survived without any short- or long-term neurological injury. This suggests either that the respiratory gas supply was misjudged or that other protective mechanisms were at work.

### 1.3. The Diver

Dc was 32 years old at the time of the accident. With a height of 1.92 m and a body mass of 95 kg, he had a BMI of 25.8 kg/m^2^ (telephone interview with JDS 2020) and thus, a body surface area of 2.25 m^2^ [10]. Before and during his time as a diver, Dc exercised three to four times a week—partially because of his annual medical examination—by running and swimming. According to his own statement, he neither took part in yoga courses nor practiced apnea diving. Without any prior experience as a scuba diver, this non-smoker became a professional diver at the age of 22. At the time of the accident, he had ten years of professional experience, with a focus on saturation diving in recent years. For the planned work assignment on the drilling template, 28 days in hyperbaric pressure, heliox breathing and 6 hrs shift work were scheduled.

### 1.4. Two Inconsistencies

1. Heliox supply. In the event of a primary gas supply failure, underwater divers must use an emergency supply of breathing gas in a bail-out bottle. In this situation, the divers are supposed to return from their workplace to the diving bell along their 27-m long umbilicals.

In this particular case, breathing gas was needed to cover 33 min from the moment the umbilical was severed to the rescue in the diving bell. For this period, Dc carried two 12-L bottles with heliox (86/14: 14% O_2_) at a pressure of 300 bar, giving a volume of 7200 L. However, at the depth of 90 m, the gas supply can only be used down to ambient pressure, which is a 10-bar filling pressure, i.e., approximately 7000 L of heliox were available. It has to be mentioned, that already at a low filling pressure of about 10 bar above the ambient pressure, thus at 20 bar, breathing resistance starts to increase due to technical reasons (Dc later reported on his breathing becoming more difficult).

Both the film and the media reported unanimously that the heliox supply at the depth of 90 m would be sufficient for 5 min only. Assuming this to be correct, 7000 L would have been breathed within 5 min, i.e., 1400 L/min at the surface. At an ambient pressure of 10 bar, a value of 140 L/min results which is physiologically entirely unrealistic.

2. Dissolved oxygen. To answer the question as to how he might have survived for the remaining 28 min, Dc suggests that enough oxygen was stored in his body as a consequence of the high ambient pressure. This concept is basically sound as Boerema et al. had shown that at elevated pressures sufficient oxygen can be physically dissolved in blood to survive for a certain time [11]. Later, a value of 4.8 mL O_2_/100 mL blood was stated [12,13] as sufficient to cover major human O_2_-demand without unloading O_2_ from hemoglobin [14].

**Figure 5:**
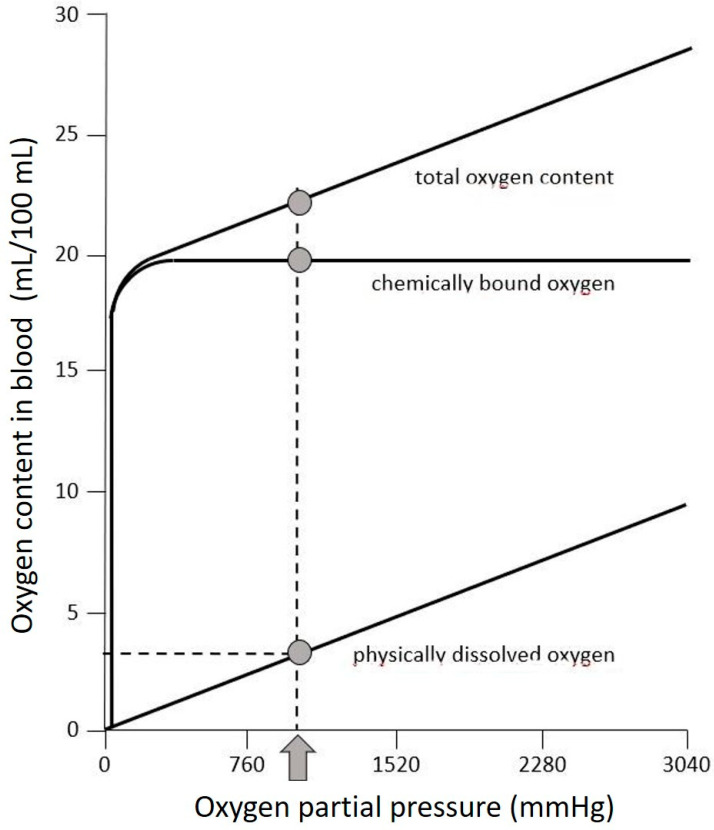
Oxygen hemoglobin dissociation curve for high O_2_ partial pressures. The pO_2_ in the present case was about 1050 mmHg, thus 3 mL O_2_/100 mL blood could physically be dissolved.

Heliox containing 14% O_2_ results in a pO_2_ of 1.4 bar at a depth of 90 m (~1050 mmHg). Thus, approximately 3 mLO_2_/100 mL blood is physically dissolved (Figure 5). With a total blood volume of 6.7 L (=95 kg body weight×7%), 200 mL O_2_ could have been additionally stored, physically dissolved in the blood. This amount would have secured survival for about one minute.

Thus, both a respiratory minute volume of 1400 L/min at depth and the suspected life supporting O_2_-storage due to the elevated ambient pressure, when limited by the blood space, are unrealistic. In the following we try to present more realistic aspects that contributed to the safe outcome.

### 1.5. Assessments

Detailed assessments are presented with respect to the heliox supply, the oxygen reserve, the hypothermia, and carbon dioxide. As they are based on data from the literature, these assessments are reliable, and thus, matched with Dc’s descriptions, they can help explain how he could survive with no injuries.

### 1.6. Heliox Consumption

To approach a realistic respiratory minute volume, Dc’s maximum ventilation volume (MVV) was calculated using Equation (1) [15]:

MVV = 86.5 − (0.522 × age) × body surface.(1)

The resulting surface MVV of 167 L/min is significantly lower than the above value of 1400 L/min. Because of the increased density and viscosity of gases at depth, the MVV decreases along with increasing depths.

**Figure 6:**
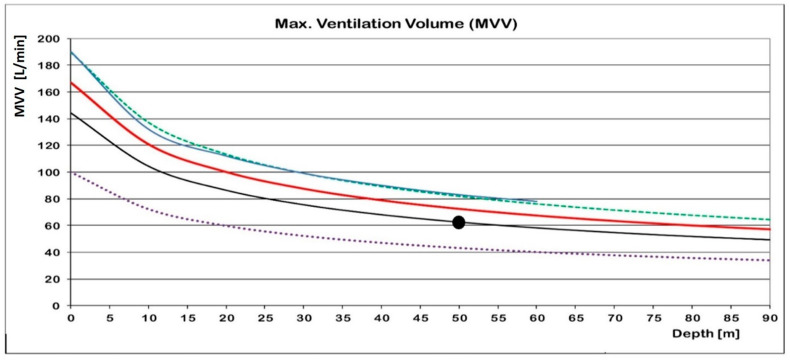
Dc’s maximum ventilation volume (MVV) with air as a function of depth (red). Similar curves are presented for untrained (dotted) and trained (dashed) divers [16], and for divers down to 60 m [17] (blue). The black line is extrapolated from a 50-m value contained in the ISO standards (EN 250) [18].

Using Equation (2) results in an MVV of 50 L/min at 90 m, if air was breathed (Figure 6, solid red curve):

MVV_(depth)_ = MVV_(surface)_/P^1/2^_(depth)_ (2)

with P = ambient pressure in bars.

Very similar curve characteristics were found in the literature and are also presented [16,17] (Figure 6). In addition, one single MVV value for the depth of 50 m was contained in the ISO standards (EN 250) [18] and was extrapolated to give a depth-depending curve (Figure 6).

Because the density and viscosity of heliox 86/14 is considerably lower than that of air, data from Figure 6 have to be adjusted to those densities.

**Figure 7:**
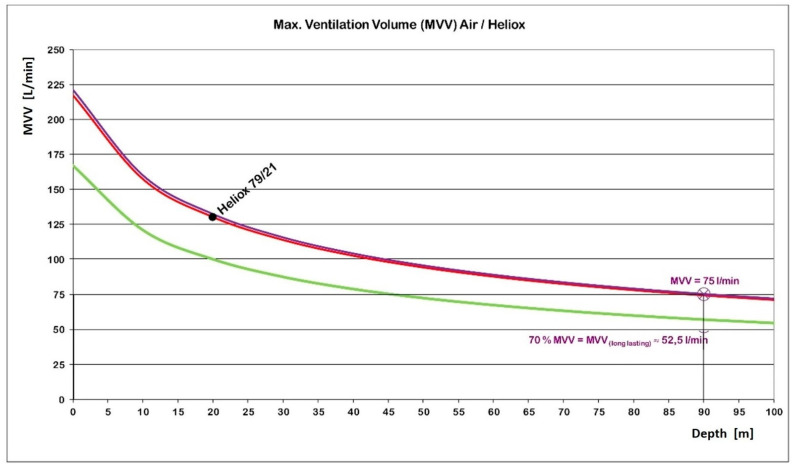
Depth-dependent maximum ventilation values (MVV) for heliox 79/21 (red). This curve was extrapolated from one single value (MVV: 130 L/min; depth: 20 m) [19] using Equation (2). The MVV for heliox 86/14 (blue) is shifted upwards somewhat, because that mix is less dense and viscous. The green curve represents respiration values during very severe work.

As yet, suitable heliox data were not found in the literature except for one single value that is valid for heliox 79/21 [19], i.e., a mix that is slightly more dense and viscous. Using Equation (2), the depth-depending MVV can be extrapolated (Figure 7), yielding a MVV of 217 L/min at the surface and 75 L/min at a depth of 90 m.

**Figure 8:**
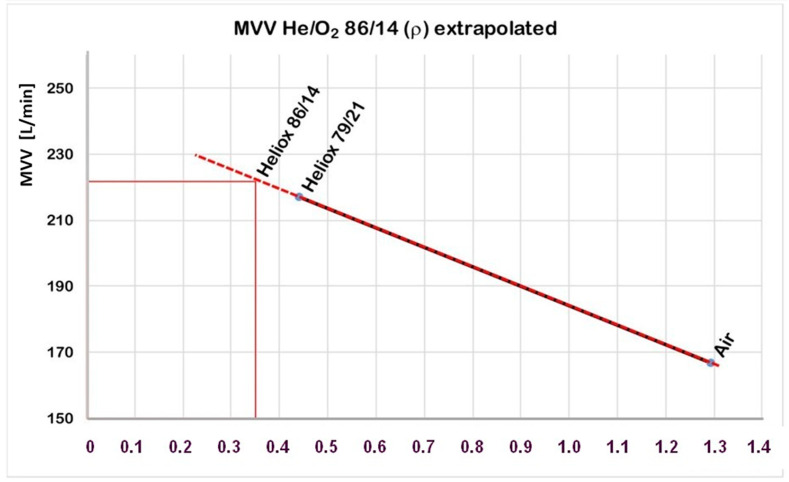
Linear extrapolation of the maximum ventilation volume (MVV) for heliox 86/14 from air and heliox 79/21 values. A surface value of 221 L/min resulted for heliox 86/14.

As a next step, it is assumed that gases follow the same physical principles in the context of respiration. Applying this assumption, the MVV for heliox 86/14 can reasonably be extrapolated from air values and heliox 79/21 values (Figure 8). giving slightly increased surface MVV values (221 vs. 217 L/min). Using Equation (2) results in a MVV of 75 l/min for the depth of 90 m (Figure 8).

It is accepted that such a MVV results from maximum voluntary hyperventilation. Not to be hit by a hyperventilation tetany, the MVV should last no longer than about 10 s. Values that can be maintained during strenuous work are, at 65–75%, considerably lower [20] and would thus range between 49 and 56 L/min (mean: 52.5 L/min) (see Figure 7).

A respiratory minute volume (RMV) of 52.5 L/min would have used up 7000 L heliox at the surface within about 130 min, i.e., within 13 min at a depth of 90 m. This period almost agrees with a statement of the bellman, who 11 min after the umbilical was severed said: ‘I have to assume that Dc does not anymore dispose of breathing gas’. Thus, the ‘official’ period of a 5-min supply of emergency heliox is very difficult to understand.

So far, it is understood that Dc breathed 525 L/min over 13 min, which would equal a value for very severe work (=290 W/m^2^) [18]. Thus, these 13 min have to be analyzed in detail.

In a later interview, Dc stated *‘…I was seized by panic …’*. This reaction is easy to understand. Immediately after the umbilical was severed, he was thrown from the platform and found himself lying on his back at the bottom of the sea. Very likely, the panic was associated with a profound psychogenic hyperventilation (=75 L/min) that could have lasted for 20 s maximum. A longer duration seems unrealistic as it would have led to hyperventilation tetany and even to unconsciousness due to a respiratory alkalosis.

Dc obviously calmed down and after a successful search for the steel construction climbed onto its 5-m high platform with much effort, pulling the remainder of the umbilical behind him (see Figure 4). This very pragmatic action can likely be attributed to Dc’s ten-year experience as a professional diver on the one hand, and to characteristics of professional divers that react more analytically towards difficult situations on the other [21]. During the 4-min ascent, normo-baric gas consumption is assumed with an upper estimate equaling 53 L/min, corresponding to very hard work. Dc stayed on the platform, became increasingly calm and thereby further reduced his respiration within the next 11 min continuously, to normo-baric 14 L/min. Later, during an interview he stated: ‘…a quiet resignation came over me’ (BBC) [22].

**Figure 9:**
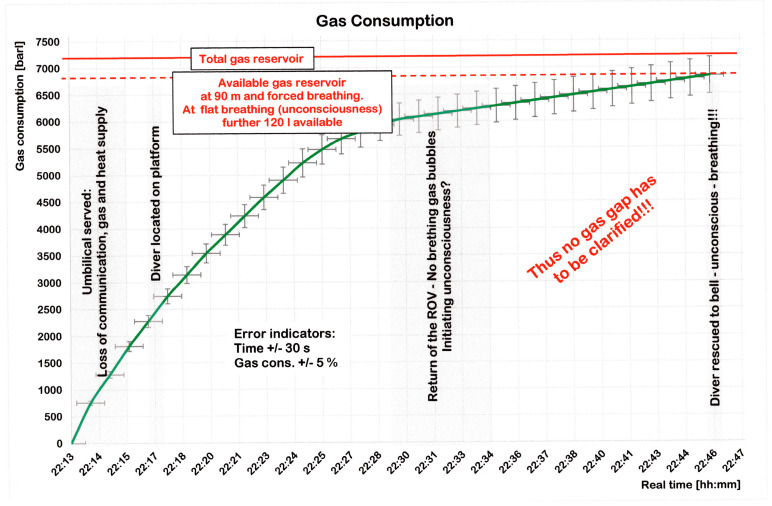
Real time gas consumption during 33 min, i.e., from the umbilical severing to the rescue in the diving bell. See also Table S1 at the end of this case report.

We suggest that the respiration slowly became even quieter and, 22 min after the umbilical was severed, Dc was detected on top of the construction by the ROV. He was waving and bubbles could be seen. However, three minutes later, Dc was motionless because, due to hypothermia, he had lost consciousness (between minute 22 and 25). In consequence, he had reduced his RMV down to 5 L/min (Figure 9), a value that produced no more visible bubbles, and that we suggest was maintained until removal of his helmet within the diving bell. As a consequence, his respiration was reported to be shallow.

### 1.7. Oxygen Reserve

Under the assumptions made so far, the heliox supply would have been sufficient for ‘healthy’ survival. If the underlying respiratory minute volumes were too optimistic, four ‘O_2_ reserves’ are pointed out, which the body could have drawn on:

1. On the surface, air contains 21% oxygen generating an O_2_-partial pressure of 0.21 bar (≈160 mmHg); assuming a hemoglobin concentration of 15 g/100 mL blood, this results in 20.1 mL O_2_/100 mL chemically bound to hemoglobin in the blood [23]. For Dc, who weighed about 95 kg, we calculated a blood volume of approximately 6.7 L, which then carries ca. 1.4 L of O_2_ bound to hemoglobin. We still have to add 0.3 L of physically dissolved oxygen, resulting in a total of 1.7 L O_2_ in blood.
2. Oxygen is also dissolved in the body’s water spaces. In the case of Chris, the water space amounts to 57 L (=60% of 95 kg). At a body temperature of 37 °C, 0.024 mL O_2_/m L /bar can be dissolved in water [24] resulting in 1.9 L O_2_ at a pO_2_ of 1.4 bar. To err on the safe side, we have not considered any increase in dissolved O_2_ that would be due to the decreased body temperature.
3. Further assuming a consumption of 0.5 L O_2_ × min^−1^, 3.6 L O_2_ would be roughly a 7-min reserve. This also means that the idea that enough oxygen was stored in Dc’s body to cover 28 min after the end of the heliox emergency supply must be dismissed.
4. We estimate a functional residual capacity (FRC) according to Equation (3) [25],

FRC = 6.98 × H + 0.017 × A − 1.734 × B − 7.511 (L)(3)

with H = height (m), A = age, B = Broca index (kg/h in cm −100). At 10 bar ambient pressure and 14% O_2_ in heliox, this results in 6.4 L O_2_ (4.6 L × 0.14 × 10).

5. The diving helmet and the neck area (Kirby Morgan 37) also contain heliox. With an assumed spherical shape and a diameter of approx. 23 cm, this results in a helmet volume of approx. 8.5 L. Subtracting a volume of 4 l for the head results in a heliox-filled volume of 4.5 L and an O_2_ quantity of 6.2 L O_2_ (=4.5 L × 0.14 × 10). Below the helmet (between the diving suit and neck), there is an almost cylindrical space. This heliox-filled area has a volume of about 1 land thus contains 1.4 L O_2_. (=1 L × 0.14 × 10). The head and neck region thus contained a total of 7.6 L O_2_. The heliox-filled area inside the rest of the suit was not likely to be available for breathing.
6. Myoglobin as an intramuscular O_2_ storage can bind 1.29 mL O_2_/g. With 6 g of myoglobin and an assumed muscular mass of 36 kg [26], Dc has a further reserve of 0.3 L O_2_. We understand that the cardiac and the skeletal muscle can also consume this relatively small O_2_ portion.

Thus, the total O_2_ reserve calculated inside the diving suit and in the ‘System Dc’ adds to about 18 L. Under resting conditions, O_2_ consumption is 4.0–4.5 mL/min per kg body weight, i.e., about 400 mL/min (=4.3 mL/min/g × 95 kg); thus, the O_2_ supply would theoretically have lasted for roughly 45 min.

### 1.8. Hypothermia

At a water temperature of 4 to 5 °C, due to the good thermal conductivity of water, a diver loses body temperature, despite a well-insulating suit. This loss alone does not lead to hypothermic conditions within 33 min (pers. data D.B. and J.D.S.).

**Figure 10:**
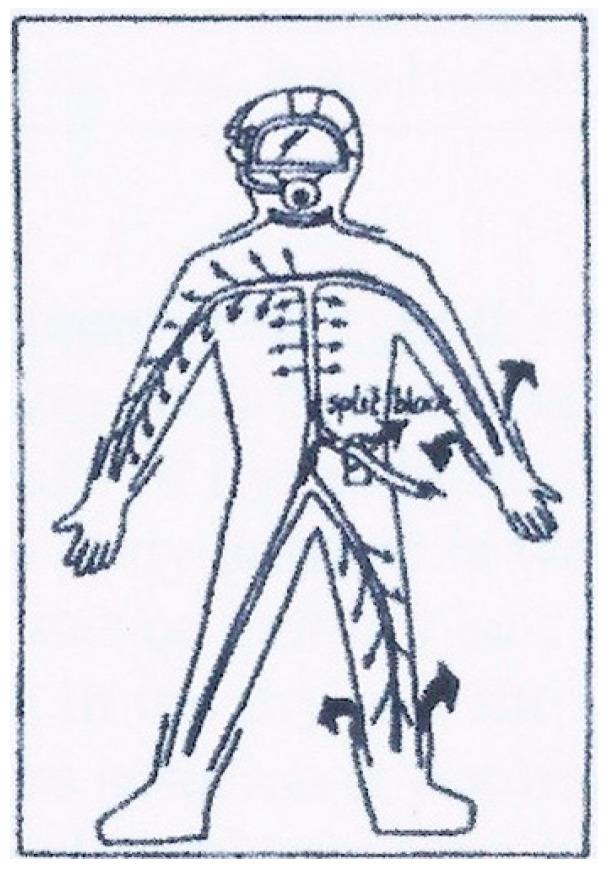
Dry suit with hot water supply. Reprinted with permission form Ref. [29]. Copyright 2004 Prof. Hans Örnhagen, SE.

However, with heliox as a breathing gas, a considerable respiratory heat loss is added, because heliox has four to five times higher thermal capacity than air, so divers must be protected with a warm water suit [27] (Figure 10). Heliox is also heated inside the suit. If the hot water supply fails, due to the high thermal conductivity five to six times higher than air, heliox in the suit cools rapidly to ambient, i.e., water temperature [28]. Losses due to heat conduction through the suit are also considered. These contribute to a fraction of ≤30% of the total heat loss over the time, compared with ≤70% for warming the breathing gas, the remainder being needed for humidification.

Within the first 15 min, Dc had already breathed roughly 6000 L of heliox. This means that, during this early phase, he had to warm up heliox of a very low temperature (~5 °C) to body temperature and to humidify the very dry breathing gas, thus heat energy was extracted from the body on a large scale. Although the upper respiratory tract and face have thermoreceptors, ‘silent cooling’ occurs because the lungs, and therefore the individual, do not notice cooling out [30,31]. Therefore, there is a drift into unconsciousness without cold shivering [32]. Based on the respiratory gas consumption, we calculated to what extent Dc would have cooled down between the severing of the umbilical and the rescue into the diving bell (=33 min).

**Figure 11:**
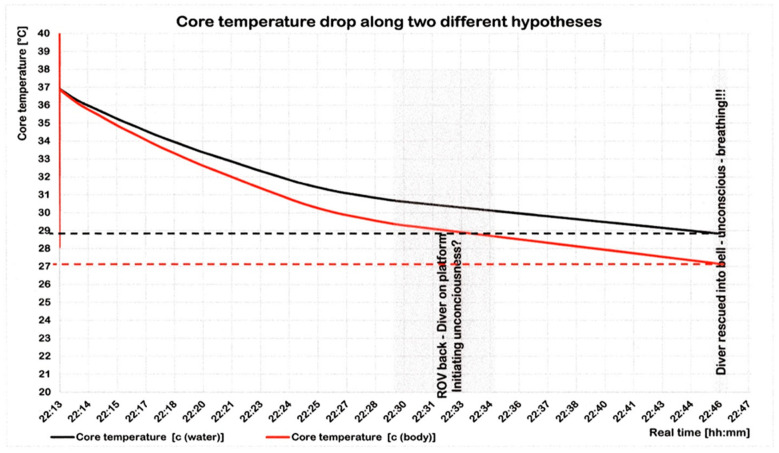
Core temperature. During 33 min after the umbilical had severed, the temperature decreased to 28.8 °C (black: hypothesis 1) and to about 27.2 °C (red: hypothesis 2).

Two hypothetical calculations were employed (Figure 11). One calculation resulted in a core temperature of 28.8 °C when assuming a heat capacity equal to water (4.18 kJ/kg × K) as a rough approximation, and a value of only 27.2 °C when assuming a heat capacity of a human, using Equation (4) [33]:

c_water_ × 0.83 = 3.47 kJ/kg × K (4)

with c = heat capacity.

The Swiss clinical staging system for accidental hypothermia defines its stage ‘2’ as ‘impaired consciousness without shivering’ (<32–28 °C) and stage ‘3’ as ‘unconsciousness’ (<28–24 °C). In concert with these two stages, Dc had been unconscious, but had not been close to stage ‘4’ ‘apparent death’ (24−13.7 °C) [34].

Classifications other than Swiss lead to similar estimates about the consequences of hypothermia. In a temperature range between 28 and 32 °C, the affected person becomes drowsy [35] or has a disturbance of consciousness [36]. As cooling continues, Dc says: *“… I felt it coming, and then…nothing.”* So he became unconscious, apparently not due to O_2_ deficiency, but due to hypothermia.

One should note that presumably all classifications apply to “classical”, i.e., externally caused hypothermia, in which the core temperature is lowered by a cold “external” temperature. These classifications were adopted here since no data or classification were found for the cooling “from the inside” likely to have dominated in the present case.

It is noted that cold is very well represented as a possible major contributor to reduced O_2_ consumption.

### 1.9. Carbon Dioxide

Any role of the effects of increased partial carbon dioxide pressures (pCO_2_) in decreasing ventilation also needs to be discussed. At a pressure of 10 bar, tissue and blood pCO_2_ should be largely enhanced following Boyle’s law. Elevation of pCO_2_ (typically 40 mmHg in arterial blood at atmospheric pressure) stimulates minute ventilation via the chemoreceptor reflex, but may result in severe breathing depression at pCO_2_-values above 80 mmHg due to progressive central sedation. Given the physiological pCO_2_, simply applying Boyle’s law would lead to critical elevations of pCO_2_ in a diver’s body, not compatible with survival even at depths of much less than 90 m.

However, some studies have shown that arterial pCO_2_ is only moderately elevated during increased ambient pressure [37,38,39,40]. Although tissue transport of CO_2_ is not entirely understood under such conditions, these measurements suggest that CO_2_ production, due to cellular metabolism, does not result in a health problem for divers under conditions of maintained lung respiration. It is remembered that well trained scuba divers and professional divers possess a lower ventilatory response to CO_2_ [41] suggesting a dominant adaptation of central CO_2_ sensitivity [42]. However, a problem could arise if breathing is severely depressed or even stops. Then, CO_2_, which is continuously produced inside the diver’s body (about 0.4 L/min), cannot be exhaled. Additional CO_2_ which must have been enhanced in tissues at such a high ambient pressure will also distribute via the arterial circulation (e.g., to the brain), because it is not eliminated any further during the pulmonary passage. Since physically dissolved CO_2_ is in equilibrium with bicarbonate, which under physiological conditions is 17.5-fold in excess of the physically dissolved fraction, the volume of distribution for CO_2_ is large, which may retard the effects of ongoing CO_2_-production. Due to missing quantitative information on the CO_2_-content of tissues under these conditions, a solid estimate of the potential harmful effects of CO_2_ production and redistribution is impossible at present. However, it appears safe to conclude that a causative role of body CO_2_ in breathing depression may only have arisen after a primary breathing depression by another mechanism, e.g., hypothermia, had occurred. Then, enhanced arterial CO_2_ levels may have contributed to a vicious cycle. Finally, it needs to be considered also that CO_2_-rebreathing inside the helmet may have resulted in breathing depression. This possibility appears rather unlikely, because clinical signs of hypercapnia were not reported, including flushed skin, bounding pulse, muscle twitches, asterixis, and rapid breathing. It is remembered that Dc was reportedly slowly breathing after his rescue. Rather, hypothermia was the driving force for reduced motor activity, including lung ventilation.

### 1.10. Helium and Neuroprotection

Although noble gases are chemically extremely inert, they display a remarkable spectrum of useful protective properties [43,44], e.g., amelioration of ischemic damage [45]. Unfortunately, their mechanisms of action are poorly understood [45], particularly the effects of helium [46]. Nevertheless, some in-vitro studies [47,48] and studies on rodent models [49,50] have demonstrated helium-induced neuroprotection. Thus, not surprisingly, breathing heliox in a rodent model of focal transient cerebral ischemia has shown beneficial effects [51]. Some of the studies investigating the use of helium/heliox as a preconditioning agent have also exhibited neuroprotective effects against hypoxia/ischemia [52,53,54].

Helium may be approved safely and effectively for clinical use in the near future [55]. Thus, if neuroprotection were provided by helium, then divers using heliox might benefit from neuroprotection in case of hypoxia/ischemia. Thus, helium may have importantly contributed to Dc’s safe and healthy survival.

## 2. Discussion

In 2012, a severe accident occurred during professional saturation diving. A film entitled ‘Last Breath’ was made about this true story and was released in the UK and Eire in cinemas from 2019 [56]. This film not only fascinates divers and staff from hyperbaric chambers, but gained additional attention from the internet because of headlines like: *‘A diver survived more than 30 min at the bottom of the North Sea after his oxygen cord was severed in an oil-rig repair job gone horribly wrong’* [57] or *‘Can you survive if you run out of air?’* [58]. According to the film [56] and the media [22,59,60], the heliox supply should have been sufficient to cover breathing for only five to six minutes. Personal data were collected from the diver, who had suffered the accident, via several telephone calls (JDS; 2021). On one such occasion, this diver granted permission to publish the data in a scientific journal. The chronology of the accident was later reconstructed in an official investigation [61].

Severing of an umbilical at a depth of 90 m presents in any case a life-threatening event for the diver. At first glance, it seems to be a miracle that Dc healthily survived. However, we made clear that there was neither a-five-minute oxygen supply nor was there enough oxygen stored in the victim’s body to make a miracle happen. Instead, six favorable circumstances may have contributed.

First. Surface-supplied professional divers carry bail-out bottles for use as an emergency supply. Here, the two bottles contained 7200 L heliox (86/14), of which up to 7000 L could be breathed at the depth of 90 m. The calculation of Dc’s per-minute breathing gas consumption, considering both the decreased viscosity of the helium mix and the pressure-related increase in viscosity, did not exhibit a breathing gas gap.

Second. Oxygen was contained in Dc’s ‘system’, consisting of the heliox volume around the head and the neck, the lungs, and the body water spaces. Our calculations gave a value of about 18 L O_2_. Even if the O_2_ consumption should have been as high as 0.5 L/min on average, this supply would have been sufficient even if there were not any external breathing gas supply for a period of 33 min. Based on the first and the second approach, we are confident that Dc did not suffer from hypoxia. After his rescue in the diving bell, he thus did not exhibit any hypoxia-induced neurological injury.

Third. At the time of the incident, Dc had already ten-years’ experience as a professional diver. Immediately after the umbilical was severed, he reports to have panicked. Apparently, he calmed down, looking for the drilling template, and climbing onto its deck for ease of location. Nevertheless, he thought ‘this will be the place where my life ends’ and he realized an increased breathing resistance and reports, *‘I felt it coming … and then: nothing’*, i.e., he lost consciousness. From this point in time, the respiratory minute volume may have been greatly reduced, likely comparable to that of with patients under anesthesia.

Fourth. The respiratory heat loss was calculated based on the heliox inhaled. Breathing this gas leads to a respiratory heat loss that is considerably higher than the conductive heat loss. At the time of Dc’s rescue, the core temperature was calculated to be 28.8 to 27.2 °C, depending on the assumptions made. In accordance with the literature, these values are highly likely to be associated with impaired or lost consciousness, respectively. As there was no deep unconsciousness, we suggest that both deep breaths given by the bellman reduced depression of the respiratory center, such that respiration normalized quickly. As a consequence, Dc soon presented fine motor skills and speaking ability.

Fifth. After relocating Dc on the drilling template, the actions of D2 and the bellman must be regarded as exemplary, in particular, the operation by D2, who finally transported the victim to the bell, where this potential catastrophe ended happily.

Sixth. One positive aspect of helium needs mentioning as this gas is inert only in a physico-chemical sense. Instead, in biological tissues it exerts neuroprotective properties that might have contributed to healthy survival in case of hypoxic conditions. Such an effect could have been even more pronounced with elevated helium partial pressures.

If these six circumstances are taken together, Dc’s healthy survival is not a miracle anymore, as it can be convincingly explained by means of reliable data. Remaining with a breathing gas supply sufficient for five minutes only would not have ended in a miracle but would have ended in death by suffocation. Nevertheless, the limit for a healthy outcome was probably very close.

Epilogue: Highly effective measures in occupational safety, in particular the considerable bail-out heliox reserve, secured healthy survival. This positive outcome is likely also to owe to the excellent diving training of a carefully selected individual, together with many years of diving routine. In addition, the rescue action of the bellman and D2, and Dc’s retrieval by the ROV operator, are suggestive for crew members with a high degree of professional qualification needed for correct operation in a hostile environment. A suggestion for further improvement would include an autonomous light for orientation in the dark and a flashlight for easier localization in a similar emergency. In addition, self-contained heating devices for divers should be considered for emergencies when the umbilical fails.

## Data Availability

Not applicable.

## Supplementary Materials

The following are available online at https://www.mdpi.com/article/10.3390/healthcare10030453/s1, Table S1: Breathing volume per minute from severing of the umbilical until the rescue into diving bell.

## References

[1] Dillig B. (2006). Dräger Safety Liefert Tieftauchanlage für Norwegisches Taucherbasisschiff “Bibby Topaz”. Presse Box.

[2] Subsea (2015). Bibby Reconstructs North Sea Diving Incident. Offshore Energy.

[3] (2016). Oxygen Content in Open Circuit Bail-Out Bottles for Heliox Saturation Diving. The Diving Medical Advisory Committee.

[4] Miller K. (2020). Bay-Tech Industries. https://baytechrentals.com/product/divers-umbilical/.

[5] Villazon Granda A. Incident Summary 08.10.12 “Bibby Topaz.” 2012. https://de.slideshare.net/arturovillazongranda/incident-summary-081012-bibby-topaz.

[6] FloatingHarbour, Co., UK (2014). Chris Lemons on Oil Rig. https://floatingharbour.co.uk/admin/resources/films/screenshot-2014-11-06-14.12.17-w1920.png.

[7] Perring R. (2019). Miracle of Diver Who Cheated Death When Stranded 300ft under with NO OXYGEN for 30 Minutes. Express.

[8] Davidson B. (2019). He was 330 Feet Deep without Anything to Breathe—and Survived. undercurrent.org. https://www.undercurrent.org/UCnow/dive_magazine/2019/330FeetDeep201906.html.

[9] Safar P., Kochanek P. (2000). Cerebral blood flow promotion after prolonged cardiac arrest. Crit. Care Med..

[10] (2005). Ergonomie der Thermischen Umgebung—Bestimmung des Körpereigenen Energieumsatzes. DIN EN ISO 8996:2005-01. https://www.beuth.de/de/norm/din-en-iso-8996/73397417.

[11] Boerema I., Meyene N.G., Brummelkamp W.H., Bouma S., Mensch M.H., Kaermans F., Hanf F.S., van Aalderen W. (1960). Leven Zonder Bloed. Ned. Tijdschr. Geneeska..

[12] Frisancho A.R. (1993). Human Adaptation and Accommodation.

[13] Malley W.J. (2005). Clinical Blood Gases: Assessment and Intervention.

[14] van Hoesen K. (1989). Hyperbaric Medicine; Chapter 78. Anesthesia Key. https://aneskey.com/hyperbaric-medicine/.

[15] Baldwin E.D., Cournand A., Richards D.W.J. (1948). Pulmonary insufficiency; physiological classification, clinical methods of analysis, standard values in normal subjects. Medicine (Baltimore).

[16] Maio D.A., Farhi L.E. (1970). Effect of Gas Density on Mechanics of Breathing. SAM-TR-70-5.

[17] Wood W.B. (1963). Ventilatory Dynamics under Hyperbaric States.

[18] (2004). ISO 8996:2004. ISO. https://www.iso.org/standard/34251.html.

[19] Linnarsson D., Ostlund A., Lind F., Hesser C.M. (1999). Hyperbaric Bradycardia and Hypoventilation in Exercising Men: Effects of Ambient Pressure and Breathing Gas. J. Appl. Physiol..

[20] Bühlmann A.A. (1981). Physiology and pathophysiology of diving. Schweiz. Z. Sportmed..

[21] Beckman T.J., Lall R., Johnson W.B. (1996). Salient personality characteristics among Navy divers. Mil. Med..

[22] Jackson D. (2019). Diver Cheated Death in North Sea Miracle. BBC News.

[23] Pittman R.N. (2011). Oxygen Transport. Regulation of Tissue Oxygenation.

[24] Schmidt R.F., Thews G., Altner H. (1990). Physiologie des Menschen.

[25] Schmidt R.F., Lang F., Heckmann M. (2017). Physiologie des Menschen: Mit Pathophysiologie: Mit Online-Repetitorium.

[26] Heinen E., Heinen E. (2019). Der Muskel-Masse-Index MMI. https://profheinen.de/koerperzusammensetzung/muskel-masse-index-mmi/.

[27] Mekjavic I.B., Golden F.S.C., Eglin M., Tipton M.J. (2001). Thermal Status of Saturation Divers during Operational Dives in the North Sea. Undersea Hyperb. Med..

[28] Barsky S.M., Tom S.N. (2003). Investigating Recreational and Commercial Diving Accidents.

[29] Örnhagen H. (2004). Thermal Problems in Diving. https://www.ornhagen.se/Thermal%20problems%20in%20diving%20050106%20Hemsida.pdf.

[30] Piantadosi C.A., Thalmann E.D. (1980). Thermal Responses in Humans Exposed to Cold Hyperbaric Helium-Oxygen. J. Appl. Physiol..

[31] Neves J., Thomas C. (2021). Fighting Exposure—Is Helium a ‘Cold’ Gas?. SDI/TDI/ERDI/PFI.

[32] Tipton M.J. (1989). The Initial Responses to Cold-Water Immersion in Man. Clin. Sci..

[33] Geigel R. (1924). Wetter und Klima: Ihr Einfluss auf den Gesunden und auf den Kranken Menschen.

[34] Musi M.E., Sheets A., Zafren K., Brugger H., Paal P., Hölzl N., Pasquier M. (2021). Clinical Staging of Accidental Hypothermia: The Revised Swiss System. Resuscitation.

[35] Pasquier M., Zurron N., Weith B., Turini P., Dami F., Carron P.N., Paal P. (2014). Deep Accidental Hypothermia with Core Temperature Below 24 °C Presenting with Vital Signs. High Alt. Med. Biol..

[36] Brown D.J.A., Brugger H., Boyd J., Paal P. (2012). Accidental Hypothermia. N. Engl. J. Med..

[37] Tibes U., Stegemann J. (1969). Das Verhalten der endexspiratorischen Atemgasdrucke, der O_2_-Aufnahme und CO_2_-Abgabe nach einfacher Apnoe im Wasser, an Land und apnoeischem Tauchen. Pflügers Arch. Eur. J. Physiol..

[38] Muth C.M., Radermacher P., Pittner A., Steinacker J., Schabana R., Hamich S., Paulat K., Calzia E. (2003). Arterial Blood Gases during Diving in Elite Apnea Divers. Int. J. Sports Med..

[39] Weaver L.K., Howe S., Snow G.L., Deru K. (2009). Arterial and pulmonary arterial hemodynamics and oxygen delivery/extraction in normal humans exposed to hyperbaric air and oxygen. J. Appl. Physiol..

[40] Bosco G., Rizzato A., Martani L., Schiavo S., Talamonti E., Garetto G., Paganini M., Camporesi E.M., Moon R.E. (2018). Arterial Blood Gas Analysis in Breath-Hold Divers at Depth. Front. Physiol..

[41] Pendergast D.R., Lindholm P., Wylegala J., Warkander D., Lundgren C.E.G. (2006). Effects of Respiratory Muscle Training on Respiratory CO_2_ Sensitivity in SCUBA Divers. Undersea Hyperb. Med..

[42] Earing C.M.N., McKeon D.J., Kubis H.P. (2014). Divers Revisited: The Ventilatory Response to Carbon Dioxide in Experienced Scuba Divers. Respir. Med..

[43] Wang Y.-Z., Li T.-T., Cao H.-L., Yang W.-C. (2019). Recent advances in the neuroprotective effects of medical gases. Med. Gas Res..

[44] Höllig A., Coburn M. (2021). Noble gases and neuroprotection: Summary of current evidence. Curr. Opin. Anaesthesiol..

[45] Winkler D.A., Thornton A., Farjot G., Katz I. (2016). The diverse biological properties of the chemically inert noble gases. Clin. Pharmacol. Ther..

[46] Weber N.C., Preckel B. (2019). Gaseous mediators: An updated review on the effects of helium beyond blowing up balloons. ICMx.

[47] Coburn M., Maze M., Franks N.P. (2008). The neuroprotective effects of xenon and helium in an in vitro model of traumatic brain injury. Crit. Care Med..

[48] Jawad N., Rizvi M., Gu J., Adeyi O., Tao G., Maze M., Ma D. (2009). Neuroprotection (and lack of neuroprotection) afforded by a series of noble gases in an in vitro model of neuronal injury. Neurosci. Lett..

[49] Aehling C., Weber N.C., Zuurbier C.J., Preckel B., Galmbacher R., Stefan K., Hollmann M.W., Popp E., Knapp J. (2018). Effects of combined helium pre/post-conditioning on the brain and heart in a rat resuscitation model. Acta. Anaesthesiol. Scand..

[50] Liu Y., Xue F., Liu G., Shi X., Liu Y., Liu W.W., Luo X., Sun X., Kang Z.M. (2011). Helium preconditioning attenuates hypoxia/ischemia-induced injury in the developing brain. Brain Res..

[51] Pan Y., Zhang H., Acharya A.B., Cruz-Flores S., Panneton W.M. (2011). The effect of heliox treatment in a rat model of focal transient cerebral ischemia. Neurosci. Lett..

[52] Li Y., Liu K., Kang Z.M. (2016). Helium preconditioning protects against neonatal hypoxia-ischemia via nitric oxide mediated up-regulation of antioxidases in a rat model. Behav. Brain Res..

[53] Heinen A., Huhn R., Smeele K.M.A., Zuurbier C.J., Schlack W., Preckel B., Hollmann M.W. (2008). Helium-induced Preconditioning in Young and Old Rat Heart. Anesthesiology.

[54] Pagel P.S., Krolikowski J.G., Pratt P.F., Shim Y.H., Amour J., Warltier D.C., Weihrauch D. (2008). The Mechanism of Helium-Induced Preconditioning: A Direct Role for Nitric Oxide in Rabbits. Anesth. Analg..

[55] Deng J., Lei C., Chen Y., Fang Z., Yang Q., Zhang H., Cai M., Shi L., Dong H., Xiong L. (2014). Neuroprotective gases--fantasy or reality for clinical use?. Prog. Neurobiol..

[56] da Costa R., Parkinson A. (2019). Der Letzte Atemzug—Gefangen am Meeresgrund. Arte. https://www.youtube.com/watch?v=JvUab7KhojI.

[57] Baker S. (2019). A Diver Survived more than 30 Minutes at the Bottom of the North Sea after His Oxygen Cord was Severed in an Oil-rig Repair Job Gone Horribly Wrong. Businessinsider.

[58] Gray R. (2019). Can You Survive If You Run Out of Air?. BBC Future.

[59] Evans C. (2019). The Last Breath: How diver Chris Lemons Survived without Oxygen for 30 Minutes on the Seabed. Inews/Film.

[60] Lemons C. (2019). About Chris. https://www.chrislemons.co.uk/about-chris.

[61] (2014). Incident Summary 08.10.12 “Bibby Topaz.”. Docomen.

