# Supplementary material for: How to Survive 33 min after the Umbilical of a Saturation Diver Severed at a Depth of 90 msw?
Source: Healthcare (Basel). 2022 Feb 28;10(3):453. doi: 10.3390/healthcare10030453 (PMC8956028; doi:10.3390/healthcare10030453)
Supplement: Supplementary file 1 [file healthcare-10-00453-s001.zip › healthcare-1541578-supplementary.pdf]

Table S1. Breathing volume per minute from severing of the umbilical until the rescue into diving bell.

Elapsed Time	Time	Breathing Volume per minute	Total Gas Consumption	Comments
[min]	[hh:mm]	[L/min]	[barL]	
0:00	22:13		0	Umbilical severed "… I was touched by panic" (MVV at amb. cond)
0:01	22:14	75	754	First minute hyperventilation "… I was touched by panic" → MVV short termed 75.4 l/min (MVV at ambient cond.)
0:02	22:15	53	1282	Very heavy work → 60 L/min
0:03	22:16	53	1810	Still very heavy work → 52.8 L/min (long term MVV!)
0:04	22:17	47	2276	Arrived at platform - calms down → 46.6 L/min (heavy work)
0:05	22:18	47	2742	Remains at 46.6 L/min
0:06	22:19	40	3142	Calming down → Gas consumption decreases
0:07	22:20	40	3542	Calming down → Gas consumption decreases
0:08	22:21	35	3889	Increasingly calming down "… a quiet resignation came over me" → 34.7 L/min
0:09	22:22	35	4236	Remains at 34.7 L/min

0:10	22:23	35	4583	Remains at 34.7 L/min
0:11	22:24	32	4903	Getting even more quiet 32 L/min
0:12	22:25	32	5223	Still 32.7 m/min – 5700 L Heliox consumed by now → progressive cooling-down!!!
0:13	22:26	25	5473	Breath becomes flatter 25 L/min
0:14	22:27	20	5673	… even flatter → 20 L/min
0:15	22:28	13	5803	Breath becomes increasingly flatter 16 L/min
0:16	22:29	13	5933	"I felt it come - and then …nothing" → 13 L/min
0:17	22:30	10	6033	Initiating unconsciousness due to core temperature drop → 10 L/min
0:18	22:31	5	6083	Ongoing unconsciousness → max. 5 L/min – Thus, ROV sees no gas bubbles
0:19	22:32	5	6133	Ongoing unconsciousness → max. 5 L/min
0:20	22:33	5	6183	Ongoing unconsciousness → max. 5 L/min

0:21	22:34	5	6233	Ongoing unconsciousness → max. 5 L/min
0:22	22:35	5	6283	Ongoing unconsciousness → max. 5 L/min
0:23	22:36	5	6333	Ongoing unconsciousness → max. 5 L/min
0:24	22:37	5	6383	Ongoing unconsciousness → max. 5 L/min
0:25	22:38	5	6433	Ongoing unconsciousness → max. 5 L/min
0:26	22:39	5	6483	Ongoing unconsciousness → max. 5 L/min
0:27	22:40	5	6533	Ongoing unconsciousness → max. 5 L/min
0:28	22:41	5	6583	Ongoing unconsciousness → max. 5 L/min
0:29	22:42	5	6633	Ongoing unconsciousness → max. 5 L/min
0:30	22:43	5	6683	Ongoing unconsciousness → max. 5 L/min
0:31	22:44	5	6733	Ongoing unconsciousness → max. 5 L/min

0:32	22:45	5	6783	Ongoing unconsciousness → max. 5 L/min
0:33	22:46	5	6833	Ongoing unconsciousness → max. 5 L/min - Rescued into the bell. He breathes!
